# Ciprofloxacin-Induced Guillain-Barré Syndrome, Posterior Reversible Encephalopathy Syndrome, Hepatotoxicity, and Syndrome of Inappropriate Antidiuretic Hormone Secretion: A Case Report

**DOI:** 10.7759/cureus.106451

**Published:** 2026-04-05

**Authors:** Khaled Zammar, Alghalya Al-Emadi, Abeer Safan, Boulenouar Mesraoua, Gayane Melikyan

**Affiliations:** 1 Department of Neurology, Hamad Medical Corporation, Doha, QAT; 2 Department of Medicine, Qatar University, Doha, QAT; 3 Department of Neurology, Hamad General Hospital, Doha, QAT

**Keywords:** ciprofloxacin, guillain-barré syndrome, hepatotoxicity, posterior reversible encephalopathy syndrome, syndrome of inappropriate antidiuretic hormone secretion

## Abstract

We present a case of a 51-year-old female presenting with abdominal pain and generalized weakness, with working diagnoses of Guillain-Barré syndrome (GBS), syndrome of inappropriate antidiuretic hormone secretion (SIADH), hepatotoxicity, and posterior reversible encephalopathy syndrome (PRES) following a five-day course of ciprofloxacin for UTI. Although PRES and SIADH have been reported in relation to GBS, hepatotoxicity has never been linked to GBS in the literature. Despite the fact that each of these entities has been described in the literature as an attributable adverse effect of ciprofloxacin, this case, to the best of our knowledge, is the first to combine all these entities following its use.

## Introduction

Ciprofloxacin is a broad-spectrum fluoroquinolone antibiotic medication. It has a wide range of therapeutic and prophylactic indications in ophthalmologic, respiratory, gastrointestinal, and genitourinary infections [1]. Similar to all antibacterial agents, reports of side effects and adverse reactions range from mild to severe and life-threatening complications [2-4]. Guillain-Barré syndrome (GBS), syndrome of inappropriate antidiuretic hormone secretion (SIADH), posterior reversible encephalopathy syndrome (PRES), and hepatotoxicity have been reported with variable incidences in the literature in relation to ciprofloxacin administration. We report a case of ciprofloxacin-induced GBS, SIADH, PRES, and hepatotoxicity.

This article was previously posted as a preprint on the medRxiv server on August 14, 2024.

## Case presentation

A 51-year-old right-handed female presented to the emergency department with three days of generalized fatigue and headache. She reported mild diffuse abdominal pain, nausea, and decreased appetite, followed by heaviness in all four extremities, which she attributed to fatigue at that time. Her initial physical examination, including vital signs and systematic and neurologic examinations, was within normal limits. Her laboratory workup showed deranged liver function tests (LFTs): alkaline phosphatase 151 U/L (35-104 U/L), alanine transaminase 521 U/L (0-33 U/L), aspartate transaminase 249 U/L (0-32 U/L), total bilirubin 78 g/L (60-80 g/L), and serum ammonia 30 µmol/L (11-32 µmol/L). Therefore, the hospital admitted her for additional testing. Her past medical history is unremarkable; she has an athletic lifestyle, is a nonsmoker, and is not an alcohol consumer.

On the second day of admission, she reported progression of the heaviness in the lower extremities and inability to walk without assistance. On follow-up neurologic examination, mental status, language, speech, and cranial nerves were unremarkable except for right nasolabial fold flattening and right deviation of the tongue upon protrusion. Her motor power was 4+/5 in both upper limbs diffusely and 4-/5 in both lower limbs diffusely. Deep tendon reflexes were 1+ in the biceps, triceps, and brachioradialis and absent in the knees and ankles bilaterally. Plantar responses were flexor bilaterally. Sensory and cerebellar examinations were unremarkable. On the same day, her laboratory workup showed stable LFTs; however, she had acute-onset hyponatremia, with sodium dropping from 133 mmol/L to 122 mmol/L (133-146 mmol/L).

**Figure 1:**
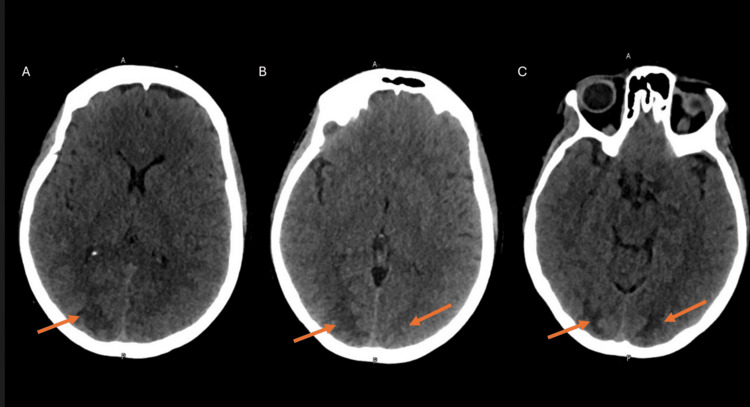
Noncontrast CT scan of the head (axial views; A-C) demonstrating bilateral, predominantly subcortical, near-symmetrical ill-defined hypodensities in the parieto-occipital regions (arrows), consistent with possible PRES PRES, posterior reversible encephalopathy syndrome

A noncontrast CT scan of the head was done and showed findings in keeping with possible PRES (Figure 1). Lumbar puncture showed mild albuminocytologic dissociation. CSF was colorless; CSF white blood cell count was 2/µL (0-5/µL), CSF red blood cell count was 20/µL (0/µL), CSF glucose was 3.56 mmol/L (2.22-3.89 mmol/L), CSF protein was 0.49 g/L (0.145-0.45 g/L), CSF IgG was 57 mg/L (0-34 mg/L), and the IgG index was 0.7 (0.3-0.6). A preliminary diagnosis of GBS was presumed, and the patient was started on an IVIG course of 2 g/kg over five days.

Retrospectively, the patient complained of some burning sensation upon micturition around 10 days prior to presentation and was treated empirically in a primary health care center with a five-day course of ciprofloxacin 500 mg twice daily. There were no investigations done at that time. The patient had no known history of recent vaccination, viral illness, or other medications.

During her hospital stay, the usual GBS care was performed, including monitoring of her forced vital capacity every four hours, which showed a decline associated with respiratory distress requiring intubation and mechanical ventilation.

**Figure 2:**
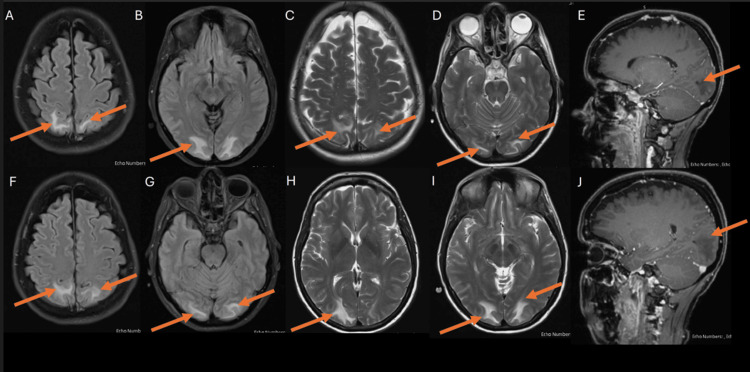
MRI of the head and spine with IV gadolinium showing bilateral cortical and subcortical areas with white matter extension, appearing relatively symmetrical, with high signal intensity (arrows) on T2-weighted imaging (C, D, H, I) and FLAIR (A, B, F, G), corresponding to low signal intensity on T1-weighted imaging, predominantly in the parieto-occipital lobes. Susceptibility-weighted imaging showed no blooming. No definite post-contrast enhancement was observed (E, J). Mild mass effect was noted on the adjacent sulci and occipital horns. Findings are consistent with PRES. PRES, posterior reversible encephalopathy syndrome

Given the above findings and clinical course, further extensive workup was done. MRI of the head and spine with IV gadolinium was ordered to rule out a central cause and showed findings consistent with PRES (Figure 2). A pan-CT scan was done for screening for tumors or masses and was unremarkable. Extensive autoimmune and infectious workups (including hepatitis screen, HIV, viral PCR, fungal infections, and tuberculosis) on serum and CSF were negative.

Hyponatremia workup showed urine osmolality of 660 mOsm/kg (150-1150 mOsm/kg), urine sodium of 112 mmol/L, thyroid-stimulating hormone of 0.73 mIU/L (0.30-4.20 mIU/L), and cortisol AM level of 579 nmol/L (138-689 nmol/L). These findings are diagnostic of SIADH and were managed accordingly with fluid input and output charting, restriction of fluid intake, and monitoring of serum sodium. Ultrasound of the liver and biliary system was unremarkable.

On the seventh day of admission, the patient began to show improvement in her motor power, which had been declining progressively to 0/5 in all extremities. This improvement continued until she was extubated and transferred to rehabilitation after three weeks in the intensive care unit. Her LFTs and sodium levels normalized within one week. On the 10th day of hospitalization, a nerve conduction study (NCS) confirmed the diagnosis of early GBS (Table 1, Table 2, Table 3, Table 4).

**Table 1:** Sensory study summary NCS was performed on the 10th day of hospitalization and showed prolonged distal peak latency in the left median sensory nerve. The left and right ulnar sensory nerve studies showed reduced amplitude. Reference ranges: median SNAP onset latency ≤3.5 ms, amplitude ≥20 µV; ulnar SNAP onset latency ≤3.1 ms, amplitude ≥17 µV; sural SNAP onset latency ≤4.4 ms, amplitude ≥6 µV Delta-O = onset latency difference; Dist = distance; lat mall = lateral malleolus; NCS = nerve conduction study; O-P amp = onset-to-peak amplitude; Stim = stimulation; Vel = velocity

Stim site	Onset (ms)	Peak (ms)	O-P amp (µV)	Site 1	Site 2	Delta-O (ms)	Dist (cm)	Vel (m/s)
Left median anti-sensory (second digit)	2.3	3.8	18.8	Wrist	Second digit	2.3	13	57
Left sural anti-sensory (lat malleolus)	2.4	3.3	17.8	Calf	Lat mall	2.4	14	58
Right sural anti-sensory (lat mall)	1.6	2.3	26.6	Calf	Lat mall	1.6	13	81
Left ulnar anti-sensory (fifth digit)	2.2	3.4	8.2	Wrist	Fifth digit	2.2	12	55
Right ulnar anti-sensory (fifth digit)	1.7	3	8.3	Wrist	Fifth digit	1.7	12	71

**Table 2:** H-reflex studies Reference range: Tibial H-reflex latency ≤35 ms (height-dependent) H-lat = H-reflex latency; L-R = left-right difference; lat norm = latency normal limit

Recording site	H-lat (ms)	L-R H-lat (ms)	L-R lat norm
Right tibial (gastrocnemius)	17.03	-	<2.0
Left tibial (gastrocnemius)	Nonrecordable	-	<2.0

**Table 3:** Motor study summary Reference ranges: median CMAP distal latency ≤4.4 ms, amplitude ≥4.0 mV; ulnar CMAP distal latency ≤3.3 ms, amplitude ≥6.0 mV; peroneal CMAP distal latency ≤6.5 ms, amplitude ≥2.0 mV; tibial CMAP distal latency ≤5.8 ms, amplitude ≥4.0 mV; motor conduction velocity: upper limb ≥50 m/s, lower limb ≥40 m/s A elbow = above elbow; ADM = abductor digiti minimi; AHB = abductor hallucis brevis; APB = abductor pollicis brevis; B elbow = below elbow; B fib = below fibula head; Delta-O = onset latency difference; Dist = distance; EDB = extensor digitorum brevis; O-P amp = onset-to-peak amplitude; Poplt = popliteal fossa; Vel = velocity

Stim site	Onset (ms)	O-P amp (mV)	Site 1	Site 2	Delta-O (ms)	Dist (cm)	Vel (m/s)
Left median motor (APB)							
Wrist	3.4	9.6	Elbow	Wrist	3.7	19	51
Elbow	7.1	7.6	-	-	-	-	-
Left peroneal motor (EDB)							
Ankle	5.5	3.6	B fib	Ankle	8	33	41
Below fibula	13.5	2.7	Poplt	B fib	2	8	40
Popliteal fossa	15.5	2.2	-	-	-	-	-
Right peroneal motor (EDB)							
Ankle	3.9	2.6	B fib	Ankle	6.3	28	44
Below fibula	10.2	1.5	-	-	-	-	-
Popliteal fossa	12.2	2.2	-	-	-	-	-
Left tibial motor (AHB)							
Ankle	3.3	6.3	Knee	Ankle	8.3	35	42
Knee	11.6	4	-	-	-	-	-
Right tibial motor (AHB)							
Ankle	3.8	5.4	Ankle	Knee	7.4	37	50
Knee	11.2	3.1	-	-	-	-	-
Left ulnar motor (ADM)							
Wrist	3.3	8.4	B elbow	Wrist	3.7	21	57
B elbow	7	5.5	A elbow	B elbow	1.4	8	57
A elbow	8.4	5.2	-	-	-	-	-
Right ulnar motor (ADM)							
Wrist	2.7	7.4	B elbow	Wrist	3.1	19	61
B elbow	5.8	6.7	A elbow	B elbow	1.4	10	71
A elbow	7.2	6.5	-	-	-	-	-

**Table 4:** F-wave studies F-wave was absent in bilateral peroneal, tibial, and ulnar nerves and prolonged in the left median nerve. These findings support the diagnosis of early GBS. Reference ranges: median F-wave latency <31 ms; ulnar <32 ms; peroneal <60 ms; tibial <61 ms AB = abductor pollicis brevis; ADM = abductor digiti minimi; AHB = abductor hallucis brevis; EDB = extensor digitorum brevis; F-lat = F-wave latency; GBS = Guillain-Barré syndrome; L-R = left-right difference; lat normal = normal upper limit of F-wave latency; M-lat = M-response latency; “-” = absent/nonrecordable

Recording site	F-lat (ms)	Lat normal (ms)	L-R F-lat (ms)	L-R lat normal	M-lat (ms)	F-lat-M-lat (ms)
Left median motor (AB)	34.57	<31	-	<2.0	-	-
Left peroneal motor (EDB)	-	<60	-	<4	-	-
Right peroneal motor (EDB)	-	<60	-	<4	-	-
Left tibial motor (AHB)	-	<61	-	<4	0.67	-
Right tibial motor (AHB)	-	<61	-	<4	0.67	-
Left ulnar motor (ADM)	-	<32	-	<2.0	-	-
Right ulnar motor (ADM)	-	<32	-	<2.0	-	-

The patient had an uneventful recovery and returned to her baseline after 10 weeks of inpatient physical and occupational rehabilitation programs. Her GBS disability score upon diagnosis was 5. At the two-month follow-up, her GBS disability score was 2, and at the six-month follow-up, it decreased to 1. All the aforementioned findings led to the diagnosis of ciprofloxacin-induced GBS, hepatotoxicity, SIADH, and PRES.

## Discussion

This case report presents a rare instance of GBS associated with PRES, SIADH, and acute liver injury following a short course of ciprofloxacin. While each of these adverse effects has been individually reported with ciprofloxacin, their simultaneous co-occurrence in a single patient has not been previously described.

Ciprofloxacin-induced GBS has been supported by pharmacovigilance data. Ali demonstrated that the reporting rate of GBS with ciprofloxacin was more than four times higher than expected, indicating a statistically significant association [2]. Popescu further documented a case of severe acute axonal neuropathy following ciprofloxacin use [4].

Ciprofloxacin-induced hepatotoxicity, although rare, can be severe and potentially fatal. While mild and transient serum enzyme elevations are relatively common, cases of clinically significant acute liver injury have been documented [5]. Orman et al. characterized the clinical and histopathologic features of fluoroquinolone-induced liver injury and suggested an immune-mediated hypersensitivity mechanism rather than a direct hepatotoxic effect [6].

SIADH following ciprofloxacin use was reported by Babar, who described a patient developing hyponatremia consistent with SIADH after ciprofloxacin initiation for a UTI. The proposed mechanism involves ciprofloxacin crossing the blood-brain barrier (BBB) and modulating γ-aminobutyric acid (GABA) and N-methyl-D-aspartate (NMDA) receptors, stimulating antidiuretic hormone (ADH) release [7].

PRES associated with ciprofloxacin was reported by Ali in a patient who developed parieto-occipital headache, hypertension, and generalized seizures on day three of ciprofloxacin treatment for pneumonia, with MRI findings consistent with PRES [1].

PRES in the setting of GBS has been reported by several authors. Van Diest et al. described a patient who initially presented with PRES manifesting as generalized seizures, followed two days later by bilateral upper limb weakness confirmed as GBS [8]. Two pathophysiologic mechanisms have been proposed for this association. First, dysautonomia in GBS can cause acute hypertension, disrupting the BBB and sympathetic innervation, particularly in the posterior circulation. Second, GBS-associated cytokine release, notably IL-2, may alter capillary permeability, disrupting the BBB and impairing cerebral autoregulation [8].

SIADH is a recognized complication of GBS. Hoffmann et al. demonstrated that SIADH can even be the presenting feature of GBS [9]. Zemke et al. reported a case in which acute hyponatremia diagnosed as SIADH developed four days after GBS diagnosis [10]. Proposed mechanisms include resetting of osmoreceptor thresholds, increased tubular sensitivity to vasopressin, and autonomic fiber dysfunction affecting stretch receptors in blood vessels and ADH release [11].

In our patient, we propose that ciprofloxacin triggered a convergent cascade of immune-mediated and neurotoxic events. The shared pathophysiologic thread likely involves immune-mediated injury: molecular mimicry or direct immune activation by ciprofloxacin may have initiated peripheral nerve demyelination (GBS) and hepatic hypersensitivity (hepatotoxicity) simultaneously. The subsequent dysautonomia from GBS, combined with ciprofloxacin’s known central nervous system penetration and GABA/NMDA receptor modulation, may have independently contributed to both PRES (via BBB disruption and impaired cerebral autoregulation) and SIADH (via inappropriate ADH release). Additionally, mitochondrial dysfunction through reactive oxygen species generation and apoptotic pathways has been implicated in fluoroquinolone neurotoxicity [5,12] and may represent a unifying subcellular mechanism contributing to multiorgan injury.

It is important to consider alternative etiologies. Sepsis is a recognized cause of PRES and hepatic dysfunction and can precede GBS through molecular mimicry following infectious triggers. However, our patient had no clinical or laboratory evidence of systemic infection at the time of presentation: blood cultures were negative, inflammatory markers were not significantly elevated, and the urinary symptoms had resolved prior to admission. Furthermore, extensive infectious workup, including hepatitis panel, HIV, viral PCR, fungal cultures, and tuberculosis screening, was negative, arguing against an ongoing infectious trigger.

Other medication-related causes were also considered. Vaccine-associated GBS was excluded, as the patient had no recent vaccination history. Immunosuppressant-associated PRES was not applicable, as the patient was not on any immunosuppressive agents. No other antibiotics or medications with known associations to GBS, PRES, SIADH, or hepatotoxicity were administered. The temporal relationship between ciprofloxacin exposure and symptom onset, the absence of alternative etiologies, and the resolution of all findings following drug discontinuation and supportive care collectively support ciprofloxacin as the causative agent.

This case has several limitations. The findings are based on a single patient, limiting generalizability. Anti-ganglioside antibodies and follow-up MRI and NCS were not performed due to financial constraints. Additionally, the retrospective format and reliance on medical records may affect data accuracy. A formal causality assessment tool, such as the Naranjo Adverse Drug Reaction Probability Scale, was not applied prospectively, though the temporal association, biological plausibility, and exclusion of alternatives support a probable causal relationship.

## Conclusions

Ciprofloxacin remains a valuable antibiotic for community-acquired and nosocomial infections. This case highlights a probable causal relationship between ciprofloxacin and the simultaneous development of GBS, PRES, SIADH, and hepatotoxicity, a combination not previously reported. Clinicians should remain aware that fluoroquinolones may trigger multisystem adverse effects through convergent immune-mediated and neurotoxic mechanisms, even in otherwise healthy patients receiving short courses for uncomplicated infections. Close monitoring of neurological status, liver function, and electrolytes during fluoroquinolone therapy, along with early recognition and systematic causality assessment when multiple adverse events co-occur, can improve patient outcomes. Prospective studies investigating risk factors and mechanistic pathways for fluoroquinolone-associated multiorgan injury in larger populations are warranted.
